# Spontaneous intra-parotid pseudoaneurysm of the external carotid artery: A rare case report due to an unfrequent disease

**DOI:** 10.1016/j.amsu.2019.05.005

**Published:** 2019-06-06

**Authors:** Omar Iziki, Sami Rouadi, Redallah Larbi Abada, Mohamed Roubal, Mohamed Mahtar

**Affiliations:** Department of Otorhinolaryngology, Head and Neck Surgery, King Hassan II University & Ibn Rochd Hospital, Morocco

**Keywords:** Pseudoaneurism, Parotid gland, Kimura disease, External carotid artery

## Abstract

Spontaneous intra-parotid pseudoaneurysm of the external carotid artery are very rare, only one case has been reported in the literature until 2019. The authors describe the second rare case of a spontaneous pseudoaneurysm of the external carotid artery which mimicked a parotid neoplasm in a young patient with pulsatile and non-fixed mass. The clinical presentation, diagnosis, and management of this condition are discussed. We report a new etiology of this rare entity that should be considered in the differential diagnosis of an atypical parotid mass.

## Hightlights

• Vascular lesions are a rare cause of parotid masses.
• Only one case of spontaneous intra-parotid pseudoaneurysm of the external carotid artery has been reported in the literature until 2019.
• The etiology of this spontaneous lesion remains unknown, this is the first time that kimura disease has been described like an etiology of this entity.
• Management of this lesion inclurdes observation, surgical excision, pressure dressings and embolization.

## 1 Introduction

This work has been reported in line with the SCARE criteria: Riaz A.Agha, Mimi R.Borrelli, Reem Farwana, Kiron Koshy, Alexander J.Fowler, Dennis P.Orgill, for the SCARE Group. The SCARE 2018 statement: Updating consensus Surgical CAse REport (SCARE) guidelines. International Journal of Surgery 2018; 60:132–136.

Parotid masses are frequent in general practice of an otorlaryngologist, with benign tumors the most common cause in adult patients. Other causes, such as malignant lesions, are less frequent. Vascular lesions are a rare but important to be known by clinicians.

The authors describe the second case in the literature of spontaneous intra-parotid pseudoaneurysm of the external carotid artery and the first due to a rare etiology.

## 2 Case report

We report a case of 22-years-old man, without past parotid inflammation, trauma or history of surgery, presented with slowly progressive and a palpable mass over the left parotid since 4 years.

**Fig. 1:**
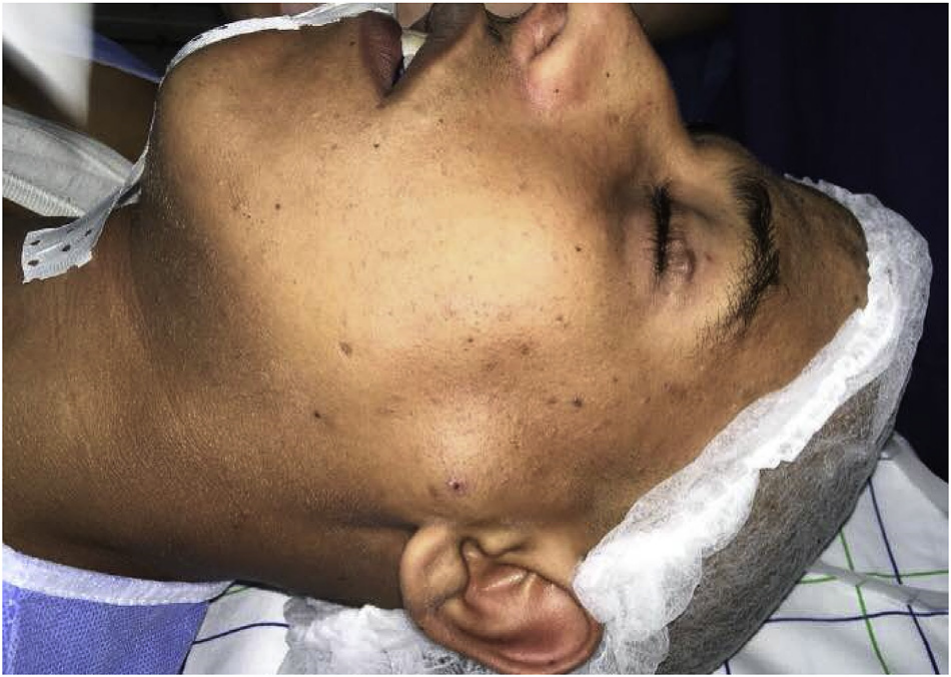
Clinical image showing the mass in the left-side.

Initial clinical examination demonstrated a palpable, pulsatile and non-fixed mass, measuring 3 cm in diameter, with small neck masses. He had no weakness of her facial nerves(Fig. 1).

**Fig. 2:**
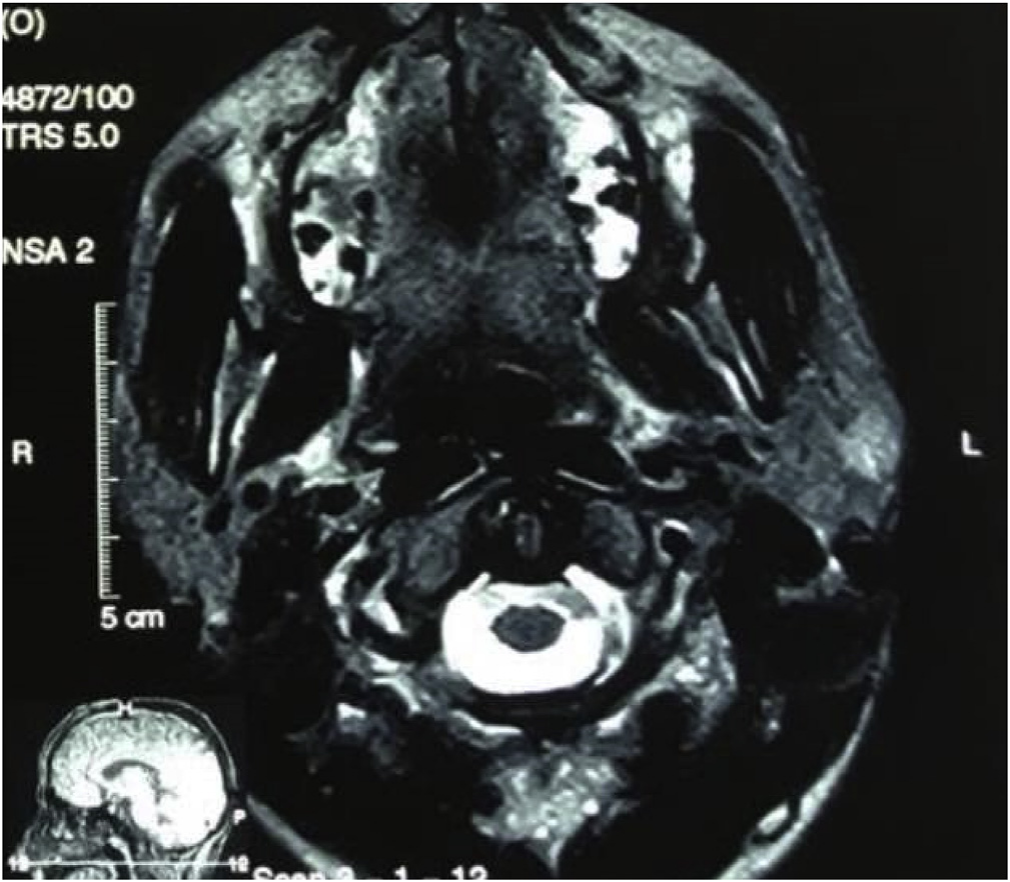
MRI T1 transverse section: visualisation of the pseudoaneurysm mimicking an intra-parotid mass.

**Fig. 3:**
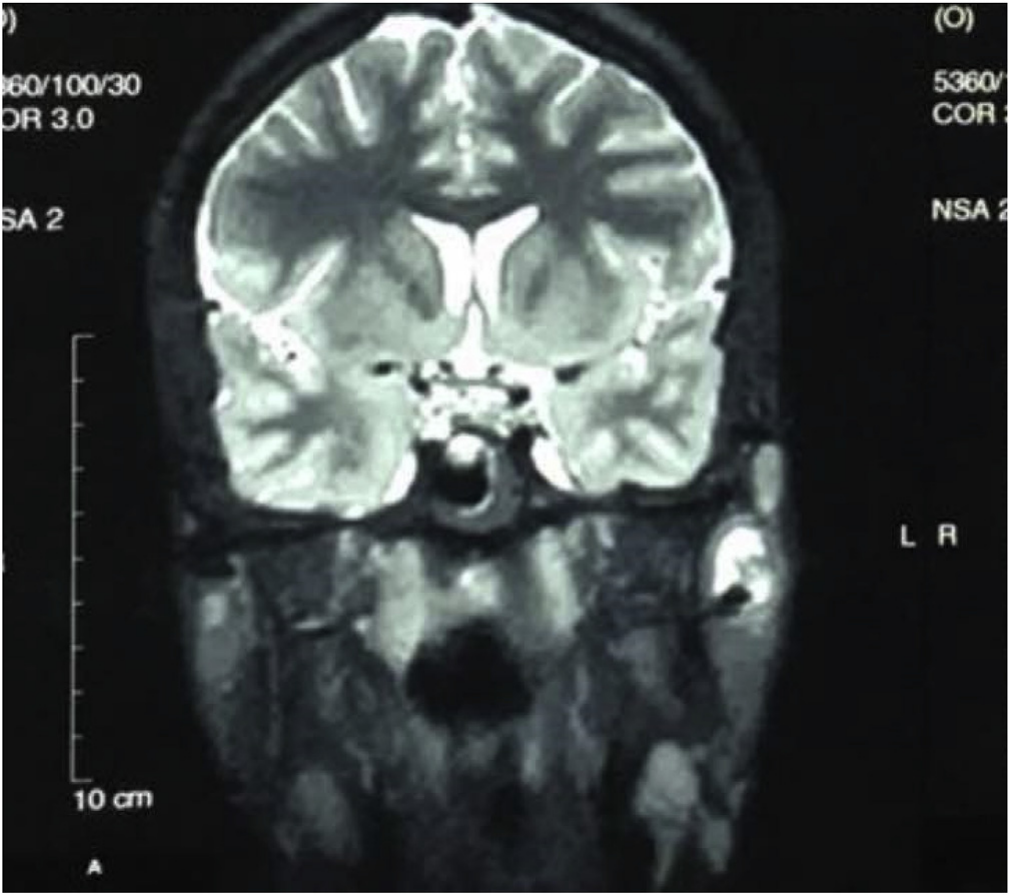
MRI T2 coronal section: visualisation of T2-hyperintense arterial blood flow (arrow) within a T2-hypointense parotid mass.

We refer patient for ultrasound examination with Doppler of the lesion that strongly suggested the vascular nature of the mass. A contrast MRI study was requested owing to the finding of clinical and ultrasound examination. MRI demonstrated a well encapsulated lesion, 20 mm in diameter, in the superficial lobe of the left parotid gland. This lesion was hyperintense T1 and T2 confirming the diagnosis of pseudoaneurysm mimicking an intra-parotid mass (Fig. 2, Fig. 3). No fine needle aspiration was performed.

**Fig. 4:**
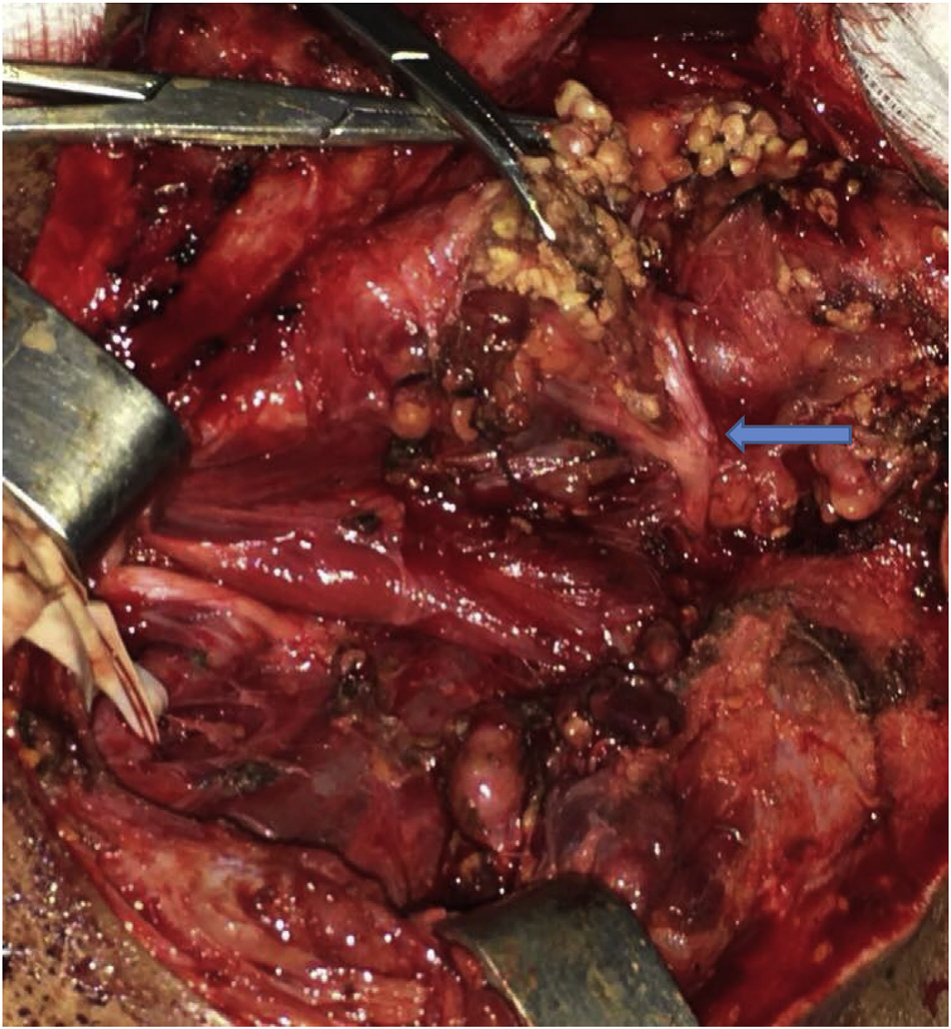
Per-operative view after superficiel parotidectomy and resection of the Pseudoaneurysm (arrow showing facial nerve).

Following discussion with the patient, the decision was to perform a surgical resection of the pseudoaneurysm starting by a superficial parotidectomy with identification and dissection of facial nerve after ligation of the facial artery. The patient was operated without incident, with a good postoperative warning (Fig. 4).

Definitive histological examination confirmed the diagnosis of pseudoaneurysm of the external carotid artery with Angiolymphoid hyperplasia and eosinophilia compatible with Kimura diseases.

The patient is undergoing regular review in the outpatient clinic at 3-month intervals during one year, and has been advised to contact the department of internal medicine for more investigations especially renal function tests, revealed without anomalies.

## 3 Discussion

Pseudoaneurysms of the extra-cranial carotid arteries are uncommon and are most commonly post-traumatic, involving the internal carotid artery [1]. Only 14 cases involving the external carotid artery or its branches have been documented in the literature until 2018, mostly occurring the superficial temporal artery and are always post-traumatic or iatrogenic [2]. There are only 5 cases involving the intra-parotid segment of the external carotid artery, in 4 of these cases a triggering factor has been identified [3]. Our patient is the second clinical case of spontaneous intra-parotid pseudoaneurysm of the external carotid artery after the case of Fernandez and al. And the first one preoperatively diagnosed [4].

The etiology of this spontaneous lesion remains unknown [2]. Woodhouse et al. attribute the cause, probably, to repeated Valsalva manoeuvres [2]. Another case reported in the literature describes rupture of a carotid vessel following this manoeuvre [2,5]. In our patient and the case of Fernandez et al. this mechanism was not observed.

In this present case, the vascular origin of parotid mass was suspected clinically, owing to the pulsatile nature of the mass, this was helpful in suggesting the diagnosis and requesting special radiological investigations. In the case of Fernandez et al. this sign was absent and the diagnosis of pseudoaneurysm of the external carotid artery was established intraoperatively [4].

Duplex ultrasound (Doppler) offers a rapid noninvasive method for detecting carotid arterial injuries, but cannot be recommended as a sole diagnostic method [1]. For Levy et al. MR imaging alone showed carotid dissection with sensitivity of 84% and specificity of 99%. But magnetic resonance angiography is more performant with sensitivity of 95% and specificity of 99% [6]. In this patient the diagnosis was confirmed by Doppler and MR imagine of the parotid region.

Management of this lesion includes observation, surgical excision, pressure dressings and embolization [2]. The decision depends on various factors: compression of adjacent structures, increasing dimensions of the lesion, associated symptoms and the patient's preference [4]. Our patient was treated by surgical resection of the pseudoaneurysm with ligation of the external carotid artery. A lymph node and nodular skin lesions biopsy was associated. The decision was made because the increase in the volume of the mass.

Clinical presentation and histopathologic examination of the vascular, lymph nodes and skin lesions confirmed the diagnosis of Kimura disease (KD) responsible for the intra parotid pseudoaneurysm.

Kimura disease is chronic inflammatory and rare disorder usually seen in young adults that most commonly presents as painless lymphadenopathy or subcutaneous masses in the head and neck region, associated with pruritus [7]. Renal diseases, especially nephrotic syndrome, and hypercoagulable state were found to be associated with the disease [8]. The diagnosis is mostly difficult, a biopsy or excision of the lesion is frequently required for definitive diagnosis [7]. The typical clinical presentation of KD in the head and neck region is a pre auricular masse [8]. However, Picard et al. report a case of an Aneurysm of the Radial Artery due to KD [9].

The optimal treatment for KD is not well established, surgical excisions have been considered as the gold standard, especially for located lesion. The use of steroids, cytotoxic agents, cyclosporin, pentoxifylline, and radiotherapy, has been documented with variable results [8].

## 4 Conclusion

Although rare, spontaneous pseudoaneurism of parotid should be concedered in differencial diagnostic of parotid masses. Our patient is the first case reported in the literature of an intraparotid pseudoaneurism of the external carotid artery due to the Kimura disease.

### Ethical approval

Written and signed consent has been obtained from the patient to publish this case.

## Funding

No sources of funding to declare.

## Author contribution

Omar Iziki: Corresponding author writing the paper.

Redallah Larbi Abada: study concept.

Sami Rouadi: study concept.

Mohammed Roubal: correction of the paper.

Mohammed Mahtar: correction of the paper.

## Conflicts of interest

All the authors have no personal or financial conflicts of interest regard this case report.

## Research Registration number

None.

## Guarantor

Omar Iziki.

## Consent

Written informed consent was obtained from the patient for publication of this case report and accompanying image.

## Provenance and peer review

Not commissioned, externally peer reviewed.
